# Dietary diallyl disulfide supplementation attenuates ethanol-mediated pulmonary vitamin D speciate depletion in C57Bl/6 mice

**DOI:** 10.1186/s40795-015-0012-z

**Published:** 2015-08-25

**Authors:** Michael L. McCaskill, Henry T. Hottor, Muna Sapkota, Todd A. Wyatt

**Affiliations:** 1 Global Environmental Health Science, School of Public Health and Tropical Medicine, Tulane University, 1440 Canal St. Ste 2100, New Orleans, LA 70112, USA; 2 Department of Environmental, Agricultural, and Occupational Health, College of Public Health, University of Nebraska Medical Center, Omaha, NE 68198-5990, USA; 3 Department of Medicine, Pulmonary, Critical Care, Sleep and Allergy Division, University of Nebraska Medical Center, Omaha, NE 68198-5990, USA; 4 Veterans Administration Nebraska Western Iowa Healthcare system, Omaha, NE 68105, USA.

## Abstract

**Background:**

Slightly more than 5 % of the United States population heavily consumes ethanol, i.e., more than 14 drinks for men and 7 drinks for women a week. Chronic ethanol consumption can result in increased liver disease, reduced recovery from burn injury, and more frequent and severe respiratory infections. Chronic ethanol over-consumption also leads to vitamin D dysmetabolism and depletion. Vitamin D is a fat-soluble pro-hormone that regulates musculoskeletal health, cellular proliferation/differentiation, and innate and adaptive immune response.

**Methods:**

In this study, C57BL/6 mice were fed 20 % ethanol in their water *ad libitum* for 7 weeks. Some mice were fed either a standard chow or a modified diet containing 0.15 μg/day of diallyl disulfide (DADS). Whole blood, lung tissue, and bronchial alveolar lavage fluid (BALF) were collected at sacrifice and analyzed for 25(OH) D_3_, 1,25 (OH)_2_D_3_, vitamin D receptor VDR, CYP2E1, and CYP27B1 levels.

**Results:**

Ethanol reduced 25(OH) D_3_ and 1,25 (OH)_2_D_3_ in lung tissue and BALF on average 31 %. The largest ethanol-mediated reduction was in the 1,25 (OH)_2_D_3_ (42 %) measured in the BALF. Dietary supplementation of DADS restored BALF and lung tissue protein of 25(OH) D_3_ and 1,25(OH)_2_D_3_ to control levels. Chronic ethanol consumption also resulted in tissue increases of vitamin D response (VDR) protein, Cyp2E1, and reductions in vitamin D-activating enzyme CYP27B1. All three of these effects were attenuated by dietary supplementation of DADS.

**Conclusions:**

In conclusion, the pulmonary metabolic disturbances mediated by chronic ethanol consumption as measured by 1,25(OH)_2_D_3_ protein levels, epithelial lining fluid, and lung tissue can be ameliorated by dietary supplementation of DADS in C57BL/6 mice.

## Background

In the United States of America, approximately 52 % of the population consume at least 12 drinks a year [1]. Slightly more than 5 % of the United States population heavily consumes ethanol, i.e., more than 14 drinks for men and 7 drinks for women a week [1]. This level of chronic consumption results in well-documented liver maladies [2, 3], as well as less obvious health-related ramifications, such as reduced success in treatment of severe burn cases [4] and more frequent incidence of severe respiratory infections [5–7].

Chronic ethanol over-consumption has been shown to interfere with the activity of several essential vitamins and nutrients, e.g., vitamin D, C, E, and folic acid. However, disruptions in vitamin D activity, via the vitamin D response element (VDRE), may have the widest range of deleterious effects [8, 9]. Chronic ethanol consumption can also have an effect on lung function. A study of ethanol consumption in young adults detailed that people who consumed more than 28 drinks per week had significantly more upper respiratory tract infections (URTIs) [10]. Chronic ethanol over-consumption has been associated with increased risk of community-acquired pneumonia [11] and increased risk of developing more severe acute respiratory distress syndrome (RR 1.59) in surgery and in hospital patient populations [12]. The lungs are particularly vulnerable to ethanol over-consumption due to the propensity of ethanol cross from the blood via the alveolar capillary bed into the alveolar spaces. Some of this unmetabolized ethanol is exhaled, however, a portion of it condenses and coats the pulmonary epithelium [13]. As an inducible metabolic pathway for ethanol metabolism, CYP2E1 creates increased amounts of oxidative stress [14] and acetaldehyde, which inhibits retinoic X receptor (RXR). The RXR is a co-activator with vitamin D response (VDR) to induce VDRE (23,46,47). We believe that extended exposure of the pulmonary epithelium to ethanol can affect VDR levels.

Vitamin D is a fat-soluble vitamin naturally present in a few foods, e.g., fatty fish, liver oils, added to others, e.g., dairy products, and available as a dietary supplement. Vitamin D is produced endogenously when ultraviolet B energy interacts with human dermal layers, which initiates in situ vitamin D_3_ synthesis from seven dehydrocholesterol [15, 16]. Because most humans can synthesize adequate levels of vitamin D from sun exposure, vitamin D is often considered to be a pro-hormone as opposed to a true vitamin [17]. In the circulatory system, vitamin D_3_ binds to the vitamin D-binding protein, travels to the liver, and undergoes the first of two necessary metabolic conversions. The first of two hydroxylation steps converts the vitamin D3 into 25-hydroxyvitamin D (25(OH)D_3_) by several phase one metabolizing enzymes such as 25 hydroxylase and CYP2R1. This version of vitamin D is still biologically inactive and is further metabolized into the active form 1,25-dihydroxyvitamin D (1,25(OH)_2_D_3_) by CYP27B1 [18]. The second of these conversions was historically thought to only occur in the kidney, but it is now widely accepted that monocytes, macrophages, and bronchial epithelial cells also can initiate CYP27B1-mediated conversions of vitamin D in their respective cellular roles [19–21]. Active vitamin D (1,25(OH)_2_D_3_) can cross membranes and act within the cell by binding to VDRs in the nucleus of effector cells and epithelia. VDRs are ligand-activated transcription factors that interact with VDRE on vitamin D-regulated genes [22, 23]. Most tissues have VDRs, including airway epithelium [22]. 1,25(OH)_2_D_3_ binding to the VDR also stimulates the activity of 1,25(OH)_2_D_3_-degrading enzyme CYP24A1 and downregulates 1,25(OH)_2_D_3_-activating enzyme, CYP27B1 [9]. This is an important feedback mechanism allowing 1,25(OH)_2_D_3_ to self-regulate circulating and tissue vitamin D levels [15]. 1,25(OH)_2_D_3_ also regulates extra-calcemic effects, such as controlling genes to regulate cellular proliferation and apoptosis; both of which are associated with a host of metabolic growth disorders including cancer and adaptive/innate immune response [24]. Vitamin D metabolic disturbances leading to low systemic and tissue levels of 25(OH)D_3_ have been associated with low levels of lung tissue antimicrobial cathelicidin/LL37, upper respiratory tract infections, and even septicemia [23, 25].

Diallyl disulfide (DADS), a lipophilic organosulfur compound found in garlic (*Allium sativum*), has been found to have chemopreventive properties in in vivo and in vitro studies [26–28]. Animal models have shown that consumption of diallyl (di and tri) sulfide can induce expression of nuclear excision DNA repair enzymes and reduce deleterious effects caused by the redox cycling of estrogen mimic diethylstilbestrol [29–31]. The chemopreventive properties of organosulfur compounds such as DADS have been partially attributed to their ability to modulate CYP2E1-mediated bioactivation of carcinogenic chemicals [32]. CYP2E1 preferentially catalyzes the oxidation of the sulfur atom to form the sulfoxide and the sulfone (DASO and DASO_2_). This final metabolism of DASO_2_ leads to the autocatalytic destruction of CYP2E1, which is primarily responsible for the chemoprotective effects of organosulfur compounds such as DADS in vivo [33].

Although DADS and other sulfur-containing compounds found in garlic and onions have shown to have efficacy in inhibiting DNA damage and attenuating chemically induce tumors in in vitro and in vivo laboratory models [34–36], human epidemiological studies have not been as conclusive [37, 38]. It is not entirely understood how chronic ethanol over-consumption can lead to severe pulmonary-related morbidity [39] and mortality [12]. However, published works by Drs. Jaqueline Kent [9] and Kartik Shankar [40] detail an ethanol-mediated reduction in renal levels of vitamin D-converting enzyme CYP27B1 and increases in renal levels of vitamin D-deactivating enzyme CYP24A1. Although the pulmonary parenchyma is functionally divergent from renal tissue, based on these studies and others, we believe that the deleterious health endpoints associated with the alcoholic lung involve ethanol’s propensity to disturb vitamin D presence in the lungs via these converting enzymes. Thus, we hypothesized that the recovery of the presence of vitamin D in an alcoholic lung environment would be beneficial. To this date, there have been no large-scale epidemiological studies evaluating chronic ethanol exposure and pulmonary vitamin D levels. We aimed to address this gap in knowledge by designing this study to investigate chronic ethanol consumption and the resulting induction of CYP2E1 effect on pulmonary vitamin D speciates. With the aims of this study, we also intended to investigate whether this modulation could be attenuated with dietary consumption of a naturally occurring organosulfur compound, diallyl disulfide.

## Methods

### In vivo model

**Fig. 1:**
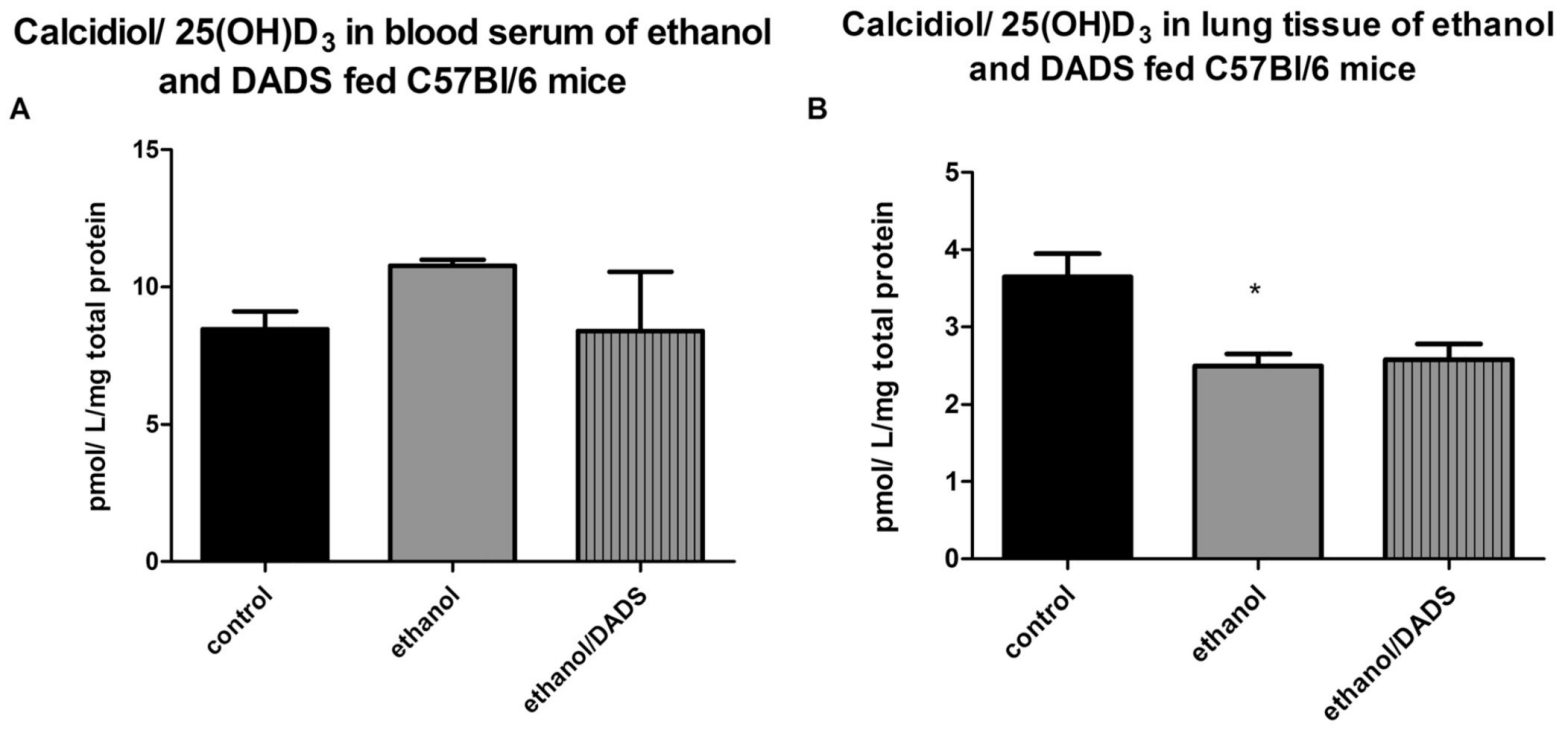
25(OH)D_3_ blood serum and lung tissue levels in 8-week ethanol- and diallyl disulfide (DADS)-fed mice. **a** Ethanol exposure resulted in statistically unchanged levels of calcidiol in the serum. **b** Lung tissue levels of calcidiol were reduced by ~50 % in the ethanol-exposed mice (*N* = 7). *p < 0.05 ethanol compared to the control

There are three in vivo experiments presented in this manuscript. Study A utilized C57BL/6 mice (female; *N* = 7) and were purchased from the National Cancer Institute (Frederick, MD, USA) at 7–8 weeks of age and housed under standard conditions. Mice were acclimated at the University of Nebraska Medical Center AAALAC-certified facility for 1 week prior to treatment exposure. C57Bl/6 mice were fed 20 % ethanol (Sigma-Aldrich, St. Louis, MO, USA) in their water via the Cook method [41] for 7 weeks including a week of increasing ethanol concentrations in their water. Mice were monitored daily and weighed weekly with no significant changes in weight observed between the two groups. For the last 6 weeks of the protocol, the ethanol-fed mice were divided into two groups fed either a (1)standard PicoLab 5058 chow or a (2)modified PicoLab 5058 diet containing 0.05 μg/1 g DADS (~0.15 μg/mouse/day). Data from this study is presented in Figs. 1 and 5.

**Fig. 2:**
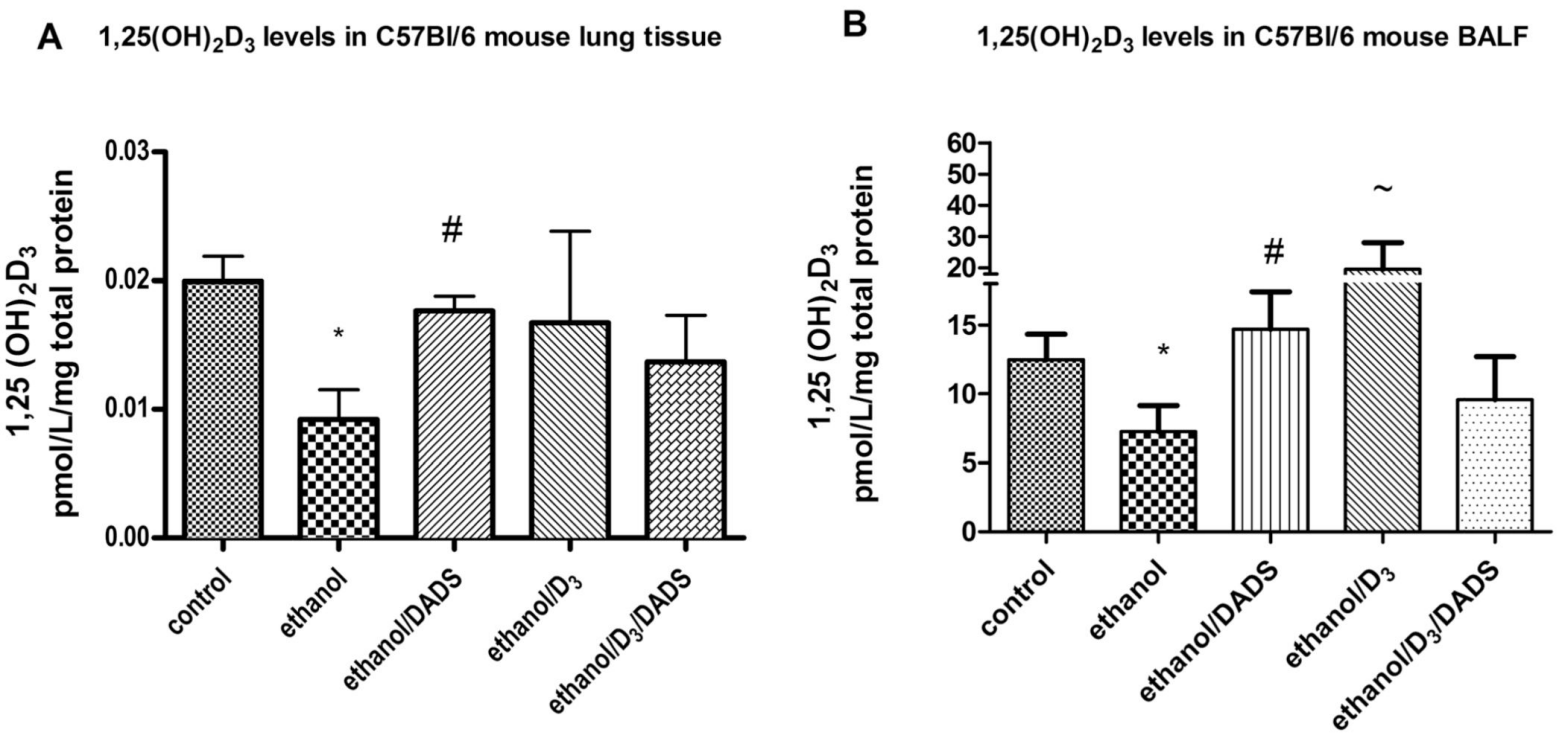
1,25(OH)_2_D_3_ levels in lung tissue and BALF of ethanol- and DADS-fed mice. Levels of 1,25(OH)_2_D_3_ in lung tissue and BALF of ethanol, 25(OH)D_3_, and DADS-fed mice. **a** Chronic dietary ethanol exposure reduced lung tissue levels of 1,25(OH)_2_D_3_ by ~50 %, and dietary consumption of DADS recovered 1,25(OH)_2_D_3_ by ~85 %. **b** Chronic dietary ethanol reduced BALF 1,25(OH)_2_D_3_ levels by ~46 %, and the co-exposure of dietary DADS recovered 1,25(OH)2D3 to control levels. Dietary exposure to higher concentrations of vitamin D3/cholecalciferol (0.25 μg/day) induces the production of 1,25(OH)_2_D_3_ to more than three times control levels. Ethanol consumption in the higher vitamin D3 reduced 1,25(OH)_2_D_3_ by ~50 %. *N* = 7 *p < 0.05 compared to control, #*p* < 0.05 compared to ethanol, ~*p* < 0.05 compared to D3 group using one-way ANOVA

Study B utilized the same methodology as study A (*N* = 7) with the addition of three treatment groups: (3) 0.083 μg/g cholecalciferol (0.25 μg/mouse/day), (4) 0.05 μg/1 g DADS (~0.15 μg/mouse/day) and cholecalciferol, (5) cholecalciferol and 20 % ethanol. DADS- and cholecalciferol-supplemented rodent chows were formulated from the same standard chow by Land O’ Lakes Purina Feed (All Points Cooperative, Sumner, NE, USA). Standard rodent chow or DADS-supplemented feed was available *ad libitum*, according to the treatment group. Data from this study generated Figs. 2 and 4.

Study C utilized the same methodology as study A (*N* = 7) with a total of four treatment groups: (1) control, (2) ethanol (20 %), (3) DADS 0.05 μg/1 g DADS (~0.15 μg/mouse/day), and (4) ethanol (20 %) and 0.05 μg/1 g DADS (~0.15 μg/mouse/day). DADS-supplemented rodent chow was formulated from the same standard chow by Land O’ Lakes Purina Feed (All Points Cooperative, Sumner, NE, USA). Standard rodent chow or DADS-supplemented feed was available *ad libitum*, according to the treatment group. Food and water consumption was documented daily. All experimental protocols were approved by the IACUC of the University of Nebraska Medical Center.

#### Mouse tracheal epithelial cell

Mouse tracheal epithelial cells were harvested from treated mice according to the methods published in [42], but briefly, tracheas were dissected and immediately placed in chilled F-12 Nutrient Ham Mixture (Invitrogen Corp.). Tracheas were then placed in pronase (1.5 mg/mL; Roche Diagnostic, Mannheim, Germany) and F-12 Nutrient mixture solution and allowed to dissociate overnight at 4 °C. The dissociated were spun in a centrifuge at 5000*g* and reconstituted in PBS and stored at 4 °C to be analyzed within 24 h.

#### Gene expression PCR array

A PCR array (Stratagene) custom gene kit was utilized to quantify the expression of CYP2E1, CYP24A1, VDR, and CYP27B1 in study group A. RT2 Real-Time SYBR Green PCR Master Mix was used for the RT2 Profiler TM PCR Array. 1225 μl of 2× SuperArray PCR master mix, 98 μl of the diluted first strand cDNA synthesis reaction product, and 1127 μl of deionized H2O were mixed. In the gene-specific 96-well plate, 25 μl of this mixture was placed in wells along with tenfold serial dilutions of this mixture. A “no reverse template control” of 1 μl of a 1:100 dilution of the total RNA and PCR master mix were also added to the 96-well plate. The thermocycler was programmed to increase temperatures to 95 °C for 10 min followed by a reduction of temperature to 60 °C for 1 min for 40 cycles. After 40 cycles, the threshold of fluorescence and the cycle at which a sample crossed the threshold was established and utilized and determine a relative genetic modulation as compared to the control treatment group.

#### Ethanol exposure

Mice in the ethanol group were given 5 % ethanol (wt/vol) to drink *ad libitum* and scaled up over 1 week (Meadows-Cook model) to 20 % ethanol (wt/vol) for 7 weeks [41, 43, 44]. Mice in the matched control group were given water from the same source without ethanol. Although this ethanol exposure model has been reported to result in an average blood ethanol concentration (BAC) level of 149 ± 47 mg/dL in the ethanol-fed mice [45], chronic ethanol consumption in this study resulted in BAC of 121 ± 42 mg/dL (data not shown).

A percentage of orally consumed ethanol diffuses from the bronchial circulation into the lumen of the airways, where some of the ethanol is then expelled during expiration. The remainder of the ethanol in the lumen condenses onto the mucosal/epithelial layer of the large airways. This can lead to localized high levels of ethanol exposure [13]. Due to this localized exposure to ethanol, we assayed lung tissue and bronchial alveolar lavage fluid to determine the effect of chronic ethanol exposure on inactive and active vitamin D availability in the lungs of ethanol-fed mice.

### 25(OH)D_3_ quantification

#### Lung tissue

To determine the level of 25(OH)D_3_ protein in the lung tissue of chronic ethanol-fed and dietary-supplemented DADS-exposed mice, lung tissue was collected at the time of sacrifice. Tissue was snap frozen in liquid nitrogen and stored at −80 °C. At the time of analysis, the tissue was thawed and ~1 g per treatment group was homogenized in lysis buffer as described in [46]. Homogenized samples (25 μl) were then used to complete the enzyme-linked immunosorbent assay (ELISA) via the immunodiagnostics systems 25(OH)D_3_ (IDS Inc, Scottsdale, AZ, USA).

#### Blood sera

Blood from C57Bl/6 mice was collected twice: prior to ethanol exposure and at the end of the 7-week ethanol treatment. Whole blood was collected, and serum was isolated using BD Vacutainer tubes (BD Biomedical, Franklin Lakes, NJ, USA). The sera from the mice were stored at −80 °C and analyzed for 25(OH)D_3_ by the University of Nebraska Medical Center clinical analytical laboratory.

### 1,25(OH)_2_D_3_ quantification

#### Lung tissue

To determine the level of 1,25(OH)_2_D_3_ present in the lung tissue of chronic ethanol-fed and dietary-supplemented DADS-exposed mice, lung tissue was collected at the time of sacrifice. The tissue was flash frozen in liquid nitrogen at the time of harvest and stored at −80 °C. At the time of analysis, the tissue was thawed, and ~1 g per treatment group was homogenized in lysis buffer as previously described [46]. A sample of 250 μl of homogenized tissue per animal was assayed for 1,25(OH)_2_D_3_ by ELISA (IDS Scottsdale, AZ, USA).

### Bronchial alveolar lavage fluid

Bronchoalveolar lavage fluid (BALF) was collected as previously described [47]. Just after completion of the ethanol exposure period, mice were euthanized by intra-peritoneal injection of pentobarbital (Nembutal; Abbott Labs, Chicago, IL, USA). Each trachea was then exposed and a cannula inserted just below the larynx. Sterile phosphate buffered saline (PBS; Gibco, Grand Island, NY, USA) was instilled (1.0 ml) into the lungs and recovered by aspiration. The 1.0 ml PBS instillation procedure was repeated three times for a total of ~3.0 ml kept separately according to the round of instillation. The first pool of the 1.0 ml of PBS was separated from the second pool and combined to recover a volume of 1.8 ml. The BALF was centrifuged at 250 *g* to collect cells. The supernatant from the first fraction of BALF fluid recovered was used to test for 1,25(OH)_2_D_3_.

### Phase 1 metabolizing enzyme assay

#### CYP27B1/CYP2E1

To determine the level of phase 1 proteins CYP27B1 and CYP2E1 in the lung tissue of chronic ethanol-fed and dietary-supplemented DADS-exposed mice, lung tissue was collected at the time of sacrifice. The tissue was flash frozen in liquid nitrogen at the time of harvest and stored at −80 °C. At the time of analysis, the tissue was thawed and ~1 g per treatment group was homogenized in lysis buffer as described [46]. CYP27B1 and CYP2E1 protein ELISA kits (USCN Life Sciences, Hubei, PRC) were used to quantify lung tissue levels of these enzymes of interest.

### Statistical analysis

Using GraphPad Prism v5.0, statistical significance of *p* < 0.05 in the experimental murine groups (*N* = 7–8) was determined by using Student’s *t* test or one-way ANOVA for multiple group statistical comparisons.

## Results

### Blood serum levels of 25(OH) vitamin D_3_ in ethanol- and DADS-fed mice

The serum levels of 25(OH)D_3_ in ethanol-fed C57BL/6 mice, when normalized to total protein, were statistically unchanged. However, a statistically significant 31 % reduction in 25(OH)D_3_ was observed in the lung tissue of ethanol-fed mice as compared to the non-ethanol-fed controls (Fig. 1).

### Lung tissue and BALF levels of 1,25(OH)_2_ vitamin D_3_ in 8-week ethanol and DADS-fed mice

When compared to the control group, 8-week ethanolfed mice displayed statistically significant reductions of 51 and 42 % in lung tissue and BALF 1,25(OH)_2_D_3_ levels, respectively. Daily dietary supplementation of DADS (~18 μg/day) restored 1,25(OH)_2_D_3_ in the lung tissue and BALF to within statistical indifference of control levels (Fig. 2).

### Chronic ethanol over-consumption modulates vitamin D receptor protein levels

**Fig. 3:**
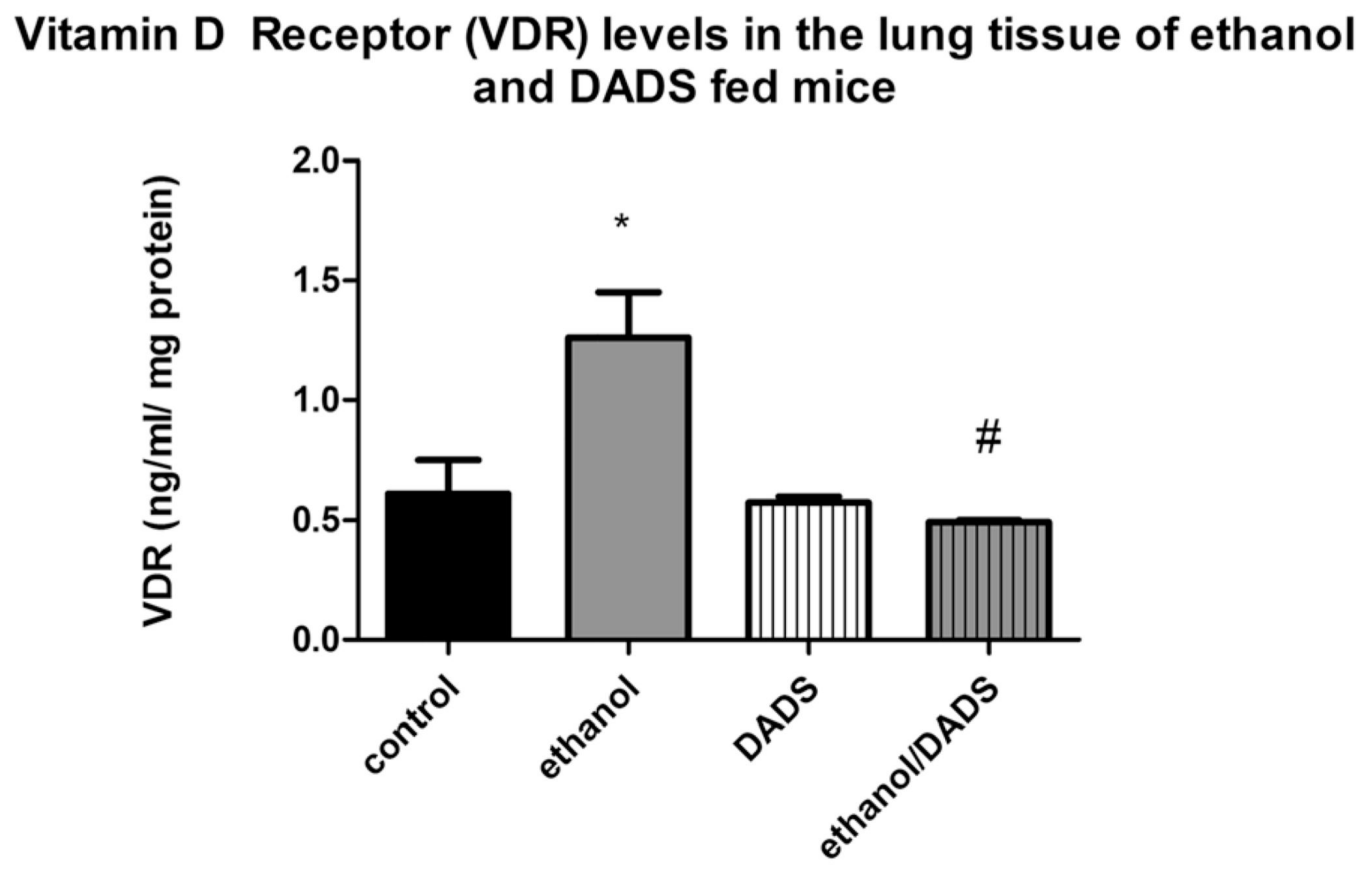
Vitamin D receptor (VDR) levels in 8-week ethanol- and DADS-fed mice. VDR levels were ~49 % higher in the lung tissue of 8-week ethanol-fed mice. The dietary supplementation of DADS completely ablated this ethanol-induced response (*N* = 7). *p < 0.05 compared to control, #*p* < 0.05 compared to ethanol group

An increase of vitamin D receptor presence (49 %) was quantified in the lung tissue of ethanol-exposed mice as compared to the non-ethanol-exposed control group. The dietary supplementation of DADS completely ablated the ethanol-induced increase in vitamin D receptor protein levels, resulting in an ethanol and DADS co-exposure vitamin D receptor profile that is statistically indifferent to the control group (Fig. 3).

### Chronic ethanol consumption and daily dietary DADS supplementation modulate vitamin D-activating phase 1 metabolizing enzyme Cyp27B1 and inducible phase 1 metabolizing enzyme Cyp2E1

**Fig. 4:**
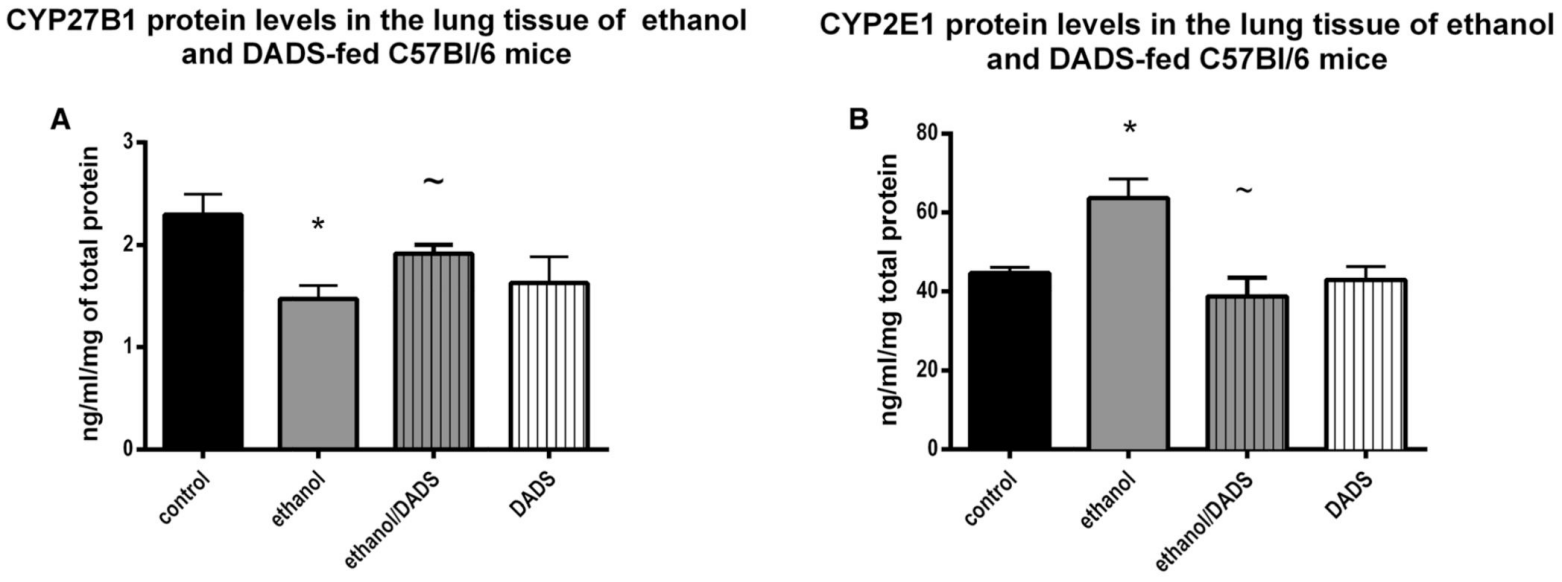
CYP27B1 and CYP2E1 enzyme levels in lung tissue of ethanol-fed mice with dietary supplementation of DADS. **a** CYP27B1 levels in ethanol-fed mice were ~35 % less than the levels in the ethanol group compared to the control. The dietary supplementation of DADS recovered this ethanol-induced reduction by 65 %. **b** CYP2E1 levels in ethanol-fed mice were statistically significantly increased by ~32 %. This upregulation of CYP2E1 protein was completely ablated by the dietary supplementation of DADS (*N* = 7). *p < 0.05 compared to control, ~*p* < 0.05 compared to ethanol group

Cyp27B1, the primary vitamin D-activating enzyme, was reduced in the lung tissue ethanol-exposed mice by 35 % as compared to the non-ethanol-exposed controls. The dietary co-exposure of ethanol and DADS resulted in a 65 % recovery of lung tissue levels of Cyp27B1. Also observed was an ethanol-mediated increase of 35 % in CYP2E1 lung tissue protein levels as compared to the non-ethanol-exposed control group. The dietary co-exposure of ethanol and DADS resulted in CYP2E1 lung tissue levels statistically indistinguishable from the control group (Fig. 4).

### Gene expression in tracheal epithelial cells of ethanol and diallyl disulfide-fed mice

**Fig. 5:**
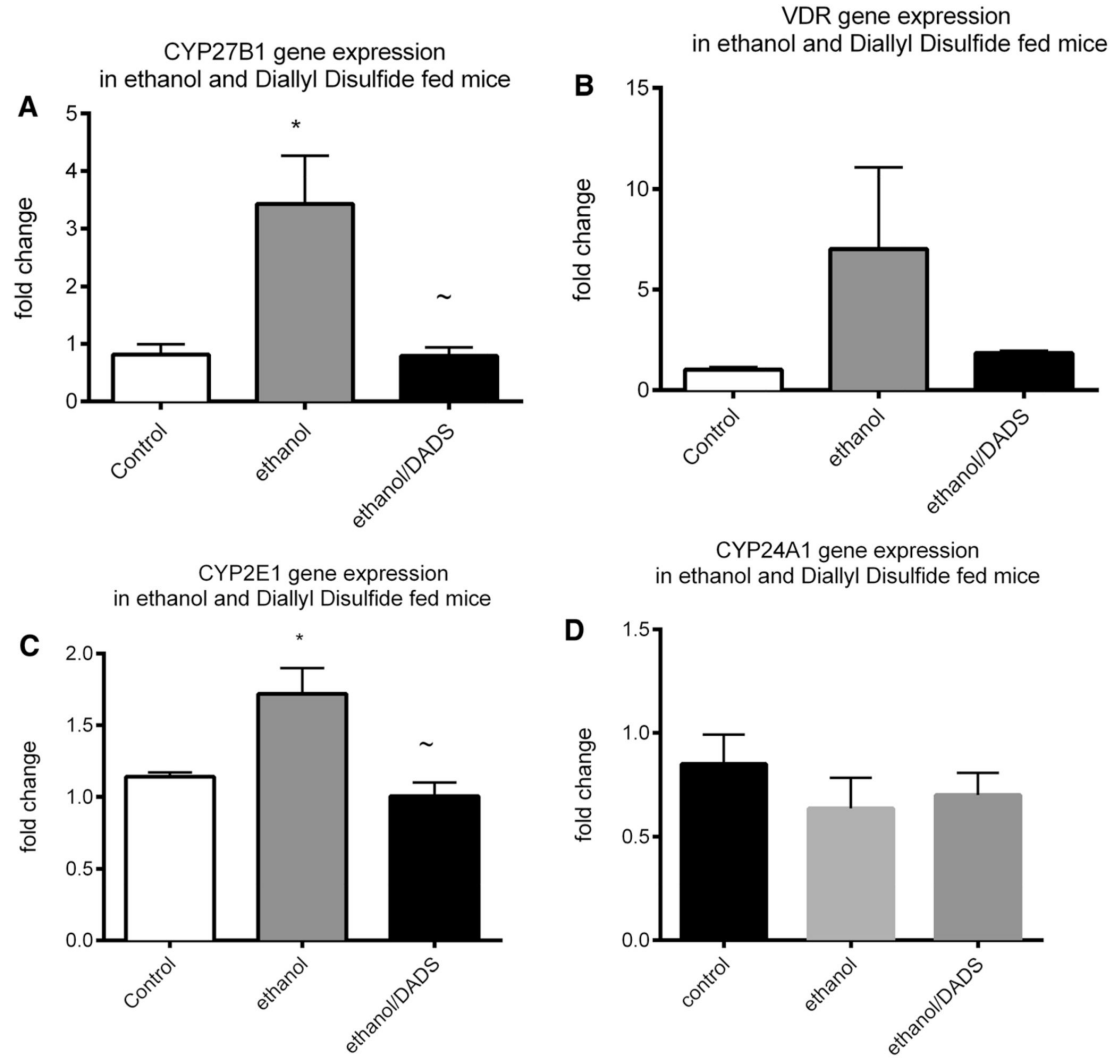
Gene expression in tracheal epithelial cells of ethanol and diallyl disulfide-fed mice. Gene expression was quantified in tracheal epithelial cells of 6-week ethanol- and diallyl disulfide (DADS)-fed mice. **a** An ethanol-mediated threefold upregulation of gene expression was observed in calcidiol-converting enzyme CYP27B1 gene expression. This upregulation was attenuated by DADS co-exposure. **b** A non-statistical ethanol-mediated sevenfold upregulation of ethanol-induced VDR gene expression was observed, and the co-exposure of DADS attenuated this upregulation. **c** A statistically significant increase in gene expression was observed in acetaldehyde-producing CYP2E1 in ethanol-fed mice that was ablated by co-exposure to DADS. **d** A slight non-statistically significant reduction in CYP24A1 gene expression was observed in the tracheal epithelial cells of chronic ethanol and diallyl disulfide-fed mice (*N* = 7; *p < 0.05 as compared to the control group, ~*p* < 0.05 as compared to the ethanol group)

Gene expression was quantified in tracheal epithelial cells of 7-week ethanol and diallyl disulfide (DADS)-fed mice. A statistically significant ethanol-mediated three-fold upregulation of gene expression was observed in calcidiol-converting enzyme CYP27B1 gene expression. This upregulation was attenuated by DADS co-exposure (Fig. 5a). A non-statistical ethanol-mediated sevenfold upregulation of ethanol-induced VDR gene expression was observed, and the co-exposure of DADS attenuated this upregulation (Fig. 5b). A statistically significant increase in CYP2E1 gene expression was observed in the ethanol-fed mice that was ablated by co-exposure to DADS (Fig. 5c). A slight non-statistically significant reduction in CYP24A1 gene expression was observed in the tracheal epithelial cells of chronic ethanol and diallyl disulfide-fed mice (Fig. 5d).

## Discussion

The association between pulmonary infections and chronic ethanol over-consumption has been well established in the medical community. Pulmonary infections have been known to occur more frequently and lead to a higher rate of mortality in people who chronically over-consume ethanol [11, 48]. Also, recent publications have presented laboratory and epidemiological data describing the role chronic alcohol over-consumption has on systemic vitamin D activation [40] and in immune and inflammatory responses [12]. The objectives of this study were to investigate the proposed mechanisms by which ethanol over-consumption reduces active vitamin D availability in lung tissue and the pulmonary epithelium lining environment (ELE). As observed in this study, chronic ethanol over-consumption did not statistically change blood serum levels of inactive vitamin D (25(OH)D_3_), but lung tissue levels of 25(OH)D_3_ were statistically lower than the control group. These seemingly incongruent results may be attributed to an abundance of dietary vitamin D precursor provided in standard rodent chow (>4000 IU, human equivalency dose) and a two compartment blood-pulmonary model acting under the influence of ethanol-induced metabolic disturbance. As ethanol is consumed and metabolized by liver enzymes, the upper airways are exposed to high amounts of ethanol for the duration of the hepatic metabolic process [13, 49]. Some extra-hepatic organs can generate and utilize phase I and II metabolic enzymes to modify xenobiotics and endogenous compounds. Often the extra-hepatic phase I metabolizing enzyme activity is utilized to activate hormones *in situ* such as vitamin D. However, under atypically high exposure conditions extra-hepatic enzymes can be temporarily inadequate to eliminate certain xenobiotics effectively. This transient state of increased exposure can result in increased xenobiotic resident times and potential organ or systemic damage. The prolonged exposure of ethanol to the bronchial/alveolar ELE of the upper airways results in reduced levels of inactive and active vitamin D in the surrounding parenchyma and ELE of ethanol-exposed mice. The bronchial ELE is the most likely location for upper respiratory infection as it is where the infectious agent meets the immune response of the lung [50]. Using BALF for a discrete assessment of vitamin D homeostasis in the pulmonary ELE, we observed a statistically significant reduction of ELE levels of 1,25(OH)_2_D_3_. As detailed in several published works [51, 52], insufficient levels of 25(OH)D_3_, and in turn 1,25(OH)_2_D_3_, can lead to an improper functioning immune response. Chronic ethanol abusers have been reported to have difficulty resisting infections, particularly in hospitals. We speculate that ethanol-mediated active vitamin D depletion could have wide-ranging effects on inflammation and innate immune response, which could partially explain statistically higher nosocomial infection incidence and mortality in hospitalized ethanol abusers [52, 53].

The mechanism behind ethanol-induced, vitamin D deficiency-mediated infection susceptibility is likely associated with an innate response. If the ELE has insufficient levels of 1,25(OH)_2_D_3_, the TLR2/1-mediated immune response could be severely impacted, resulting in decreased infectious agent clearance and increased infection severity [54–56]. We observed in our study that (Fig. 2b) chronic ethanol consumption is effective in reducing pulmonary levels of 1,25(OH)_2_D_3_ at physiologically relevant dietary vitamin D_3_ levels. It may be possible that this observation is mediated by the alveolar or bronchial epithelial cells in the pulmonary system. Our laboratory is actively investigating these potential cellular drivers of ethanol-mediated reductions in pulmonary levels of 1,25(OH)_2_D_3_.

According to our observations and supported by previously published work [9] ethanol at least partially exerts its 1,25(OH)_2_D_3_-depleting effect via the inhibition of CYP27B1. CYP27B1 is the primary metabolizing enzyme that converts inactive vitamin D into active vitamin D—1,25(OH)_2_D_3_. The observed statistically significant reduction in pulmonary levels of CYP27B1 juxtaposed against the increase in pulmonary levels of CYP2E1 in chronic ethanol over-consuming mice leads us to postulate that the metabolic fate of these enzymes may be intertwined. Interestingly enough as the protein levels of CYP27B1 were lower in the ethanol-exposed group (Fig. 4a), gene expression of CYP27B1 was modulated up threefold (Fig. 5a). We believe that the reduction of CYP27B1 in the lung tissue of chronically ethanol-fed mice and comparatively lower 1,25(OH)_2_D_3_ observed in the lung tissue and BALF of ethanol-fed mice resulted in a physiological, genetic response to initiate CYP27B1 gene expression to maintain active vitamin D homeostasis. We believe similar mechanisms are influencing the observed VDR gene (Fig. 5b) and protein (Fig. 3) upregulation observed in the chronically ethanol-fed group. As pulmonary levels of 1,25(OH)_2_D_3_ are reduced by chronic ethanol consumption, to maintain homeostasis, VDR gene expression is upregulated along with the increase of VDR presence. VDR upregulation in response to depleted vitamin D levels has been detailed in other published work and appears to be a normal homeostatic response [57]. As mentioned previously, dietary supplementation of DADS reversed the observed ethanol-mediated gene expression effects.

When ethanol-induced CYP2E1 presence was inhibited by the dietary supplementation of DADS, pulmonary active vitamin D—1,25(OH)_2_D_3_, and CYP27B1 proteins levels recovered. This observation also leads us to infer that it is possible that CYP2E1-mediated ethanol metabolism and/or metabolic intermediates are negative regulators of CYP27B1 function leading to reduced 1,25(OH)_2_D_3_ levels. This theory is supported by published work [58] as well as the observed lack of efficacy of DADS supplementation in attenuating ethanol-mediated reductions in lung tissue 25(OH)D_3_ as compared to the observed efficacy of DADS in recovering lung tissue levels of 1,25(OH)_2_D_3_.

## Conclusions

The literature is replete with articles describing ethanol’s ability to induce CYP2E1 [14, 59, 60]. However, we have confirmed that this induction can be attenuated in the lung tissue of ethanol-fed mice by dietary consumption of DADS (~0.18 μg/day). The ability of DADS to attenuate the ethanol-induced reduction of 1,25(OH)_2_D_3_ in tissue and BALF could lie in its capacity to inhibit CYP2E1 activity and ultimately, the genesis of CYP2E1-mediated reactive oxidative metabolites, such as malondialdehyde, 4-hydroxynonenal, and hydroxyethyl radical [61, 62]. Future research is warranted to evaluate this possibility.

Not only is proper 1,25(OH)_2_D_3_ function essential for effective defensin and cathelicidin/LL-37 activity in infection resistance [21, 63, 64] but also 1,25(OH)_2_D_3_ is critical to macrophage maturation, phagocytic ability, and general immune response [54, 65]. Understanding the mechanisms driving ethanol-induced pulmonary vitamin D dysmetabolism could not only help alleviate the high morbidity and mortality associated with respiratory infection rates in chronic ethanol over-consumers [52, 53] but also possibly provide general prophylaxis options for immunocompromised patients in hospital settings.
